# Odors from phylogenetically-distant plants to Brassicaceae repel an herbivorous Brassica specialist

**DOI:** 10.1038/s41598-019-47094-8

**Published:** 2019-07-23

**Authors:** Chase A. Stratton, Elisabeth Hodgdon, Cesar Rodriguez-Saona, Anthony M. Shelton, Yolanda H. Chen

**Affiliations:** 1 Department of Plant and Soil Sciences, University of Vermont, 63 Carrigan Dr, Burlington, VT 05405 USA; 2 Department of Entomology, Rutgers The State University of New Jersey, 96 Lipman Dr, New Brunswick, NJ 08901 USA; 3 Department of Entomology, Cornell University, New York State Agricultural Experiment Station, 630 West North St, Geneva, NY 14456 USA

**Keywords:** Agroecology, Chemical ecology

## Abstract

Specialist insect herbivores are constrained by highly specific odor recognition systems to accept suitable host plants. Given that odor recognition leads specialist insects to accept a limited range of plants, we hypothesized that phylogenetically distant plants produce odors that are physicochemically different from host odors and would be less attractive or even repellent to a specialist herbivore. We tested this hypothesis by examining behavioral and ovipositional responses of swede midge (*Contarinia nasturtii*, Diptera: Cecidomyiidae), a specialist of brassicas, to broccoli sprayed with non-host essential oils. Specifically, we asked: (1) How do essential oils from different plant species influence host-seeking and oviposition behaviors of swede midge? (2) Do odors from non-host plants that are not phylogenetically related or physicochemically similar to host plants affect host-seeking or ovipositional behavior of swede midge? In oviposition assays, we found that non-host odors varied in their ability to modify female midge behavior and that phylogenetic relatedness was negatively correlated with larval density. In y-tube assays, we found that female midges most frequently avoided non-host odors that were more similar to brassica odors. Females were less likely to oviposit on or choose any treated host plants, but particularly avoided garlic, spearmint, thyme, eucalyptus lemon, and cinnamon bark treatments. Overall, we found that plant phylogenetic relatedness and odor similarity are related to repellency. Therefore, altering the diversity of plant odors by explicitly accounting for plant phylogenetic distance and odor similarity, relative to host plants, may be an important, underexploited tactic for sustainably managing challenging pests.

## Introduction

The majority of phytophagous insects are specialists^1^ that use volatile cues to find and accept their host plants^2–4^. For most specialist herbivores, females recognize their plant hosts using visual cues^5–7^ and volatile organic compounds (VOCs)^8–13^. Conversely, specialist herbivores have frequently been found to be repelled by nearby non-host plants^14–16^. Repellent plants cause insects to show directed movement away from an odor source without physical contact^17^. However, the relationship between non-host plants and repellency is unclear.

Although some insect herbivores are clearly repelled by non-host odors^18–20^, there is still limited understanding on how interspecific variation of plant odors influences host-seeking and acceptance of specialist herbivores. Plant-based compounds that repel herbivores could be a valuable tool for pest management, but there are few guidelines available on how to systematically identify plant species that are repellent to specific insect pests. In fact, many previous studies on herbivore repellency selected highly aromatic plants without mentioning specific criteria for their selection, which may represent a purely ‘guesswork approach’ to identifying repellent odors for insects^21^. The complexity of plant compounds and the specificity of insect olfaction makes it difficult to predict which compounds will repel a particular herbivore^18,20,22^.

Odor recognition is highly precise and odors that repel one insect may not necessarily repel another^10^. If an odor does not bind to a specific insect receptor, it will not cause a specific effect (e.g. repellency) on the insect^23^. As reviewed by Deletre *et al*. (2016), a range of mechanisms could cause repellency. Non-host odors can mask the recognition of host odors by physically disrupting the reception of host compounds, effectively cancelling the intended effect. For example, the common insect repellent DEET (*N*,*N*-diethyl-*meta*-toluamide) masks odor recognition^24^. Regardless of the mechanism leading to repellency, the outcome is consistent – damage is reduced or does not occur. A more thorough understanding of repellency should improve ecological pest management through the development of target-specific compounds with minimal risk to the environment^21,25,26^.

Plants defend against herbivory through a wide range of defensive strategies^27,28^, resulting in a vast arsenal of defensive compounds [e.g. alkaloids, terpenoids, and phenolics^29–31^] and behaviorally active volatiles^32,33^. Furthermore, insects can detect and avoid plants using sensory mechanisms including odorant binding proteins and olfactory receptors^16,20,34,35^. While insect antennae detect volatiles in flight, tarsi and abdominal segments contain receptors that provide additional checks for plant chemistry after landing^36^. There is very little information on how to identify repellent odors. Testing how insect specialists behaviorally respond to increasingly phylogenetically distant non-host plants could provide a better understanding of how plants with non-preferred chemistry influence host-seeking and subsequent ovipositional behavior of specialist herbivores.

Identifying plant compound(s) that repel a specialist requires behavioral trials^17,21,25^. Chemical reactions are complex, and the physical/chemical properties (hereafter called physicochemical properties) of compounds dictates their interactions and biological activity. For example, if an olfactory receptor contains three binding pockets, the behaviorally relevant ligand can presumably have three binding domains^37–39^. Since an insect only detects an odor if it binds to a receptor with a specific biological structure^11^, the degree of physicochemical similarity of non-host odors (volatilized from essential oils) to a specialist’s host odors may be predictive of repellency.

Essential oils allow rapid screening of non-host compounds for their repellent properties. Their use in agriculture spans decades of research and they are generally considered safe for the environment^17,21,25,40–44^. For example, many species within Lamiaceae, the mint family, have been tested for their repellent properties because the group is highly diverse in volatile organic compounds^41^, but also because the compounds are frequently used in commercial products consumed by humans^45^. Odors vary greatly among angiosperms, so essential oils from many plant families should be considered when testing repellency.

Swede midge (*Contarinia nasturtii*; Diptera: Cecidomyiidae) is a pest of brassicas causing significant losses in Ontario and Quebec, Canada and in the northeastern United States^21^. Losses are most severe in heading brassicas (cauliflower and broccoli) because larvae feed on the developing meristem, with severe damage causing a complete loss of the marketable crown^46,47^. Because we have found that a single swede midge larva can result in marketable losses, management strategies should focus on preventing oviposition^48^. Plant-based repellents could be a valuable tool to manage swede midge, especially in organic systems that lack effective insecticides^48^. Given that swede midge specializes on *Brassica oleraceae*, their antennae are tuned to isothiocyanate groups that volatilize off plant tissue following glucosinolate-myrosinase reactions^49^. Therefore, swede midge may be repelled by all, some, or no odors from other plant families.

We tested how swede midge female oviposition varies on host plants treated with essential oils from increasingly phylogenetically distant non-host plants and odors of varying physicochemical similarity. We studied midge behavioral and ovipositional responses in no-choice, choice assays, and olfactometer choice tests. We predicted that less related plants and less similar odors are more likely to repel gravid females. No-choice assays provide a method to screen odors for their ability to repel herbivores from host plants^17^. On the other hand, choice assays are needed to understand the relative preference between non-host odors and host odors, which may be more predictive of choices in the field^46,47^. Furthermore, olfactometer assays separate the insect from contacting the odor source, thereby specifically testing host-seeking behavior. Here, we tested 18 plant essential oils from 10 plant families for their ability to repel gravid midge females from host plants using no-choice and choice assays in the lab and 15 essential oils using an olfactometer. Specifically, we asked: (1) How do larval infestations of swede midge vary on broccoli treated with different essential oils? (2) Do essential oils alter host seeking behavior of swede midge? (3) What is the relationship between phylogenetic relatedness of non-host plants to broccoli and repellency? And, (4) Does the physicochemical similarity of non-host odors to brassica volatiles influence their ability to repel swede midge? If phylogenetic relatedness and physicochemical similarity are correlated with repellency, we expected a negative relationship between these values and the behavioral data (i.e., the number of larvae found on treated plants and the number of insects avoiding treated plants in the olfactometer).

## Methods and Materials

### Plant production

Broccoli (*B*. *oleracea* group Italica ‘Packman,’ Harris Seeds, Rochester, NY, USA) plants were seeded in the University of Vermont (UVM) greenhouse in Burlington, VT in 128-cell trays (Greenhouse Megastore, LLC., Danville, IL) using Fafard^®^ 3B potting mix (Agawam, MA, USA). After 4 weeks, we transplanted seedlings into 10 cm square pots, where they grew at 21 °C and 45% RH, with a 16 L:8D photoperiod cycle until 6–8 true leaves had formed. Plants were fertilized twice weekly using 5-5-5 (N-P-K) fertilizer diluted per label directions.

### Colony Rearing

We maintained a colony of swede midge on cauliflower *B*. *oleracea* group Botrytis ‘Snow Crown’ (Harris Seeds, Rochester, NY), using a protocol described in Stratton *et al*. (2018). While we reared the midge on cauliflower for the large bud size, swede midge equally prefers cauliflower and broccoli^46^. Briefly, we placed 8–10 week- old plants into the oviposition cages, where plants were exposed to newly-emerging adults. The cauliflower plants were exposed to adults for 1 d and then moved to additional cages where larvae developed for ~14 d at ~25 °C. After larvae matured, the apical meristems were cut 3 cm below the crown and then inserted into the soil of the same pot to help 4^th^ instars reach the soil for pupation. The pots with pupating larvae were returned to the ovipositional cages, where adults emerged and mated ~14 d later^50^.

### Essential oils

We chose a diverse sampling of plant essential oils (Table 1), based on commercial availability (Bulk Apothecary, Streetsboro, Ohio) and their phylogenetic distance to Brassicaceae on the angiosperm phylogeny^51^. We ensured that the essential oils were extracted using steam distillation, a method known to extract the low-weight volatiles^21^. We applied 1% dilutions of essential oils in distilled water (concentration most frequently used in agricultural essential oil products) to meristems and leaves of experimental broccoli (15 sprays per plant; 15 mL/plant) using separate handheld spray bottles (Sprayco Consumer Products, Livonia, Michigan) for each odor treatment. In six cases, essential oils had a phytotoxic effect on the broccoli plants, causing damage ranging from light brown scarring to complete defoliation. We recorded the visual phytotoxic effects for each essential oil and used this qualitative data to restrict our recommendations for agricultural use. We also performed additional statistical tests with phytotoxic treatments excluded to ensure that the relationships between phylogeny, physicochemistry, and swede midge behavior were due to the odors rather than phytotoxicity caused by the essential oils (see Supplementary Materials).

**Table 1:** Common names, species, family, phylogenetic distance (PD), and physicochemical similarity (PS) values of essential oils compared to broccoli odors, used in no-choice and choice assays on swede midge (*Contarinia nasturtii*).

Plant	Species	Family	PD^a^	PS^b^
Peppermint*	*Mentha x piperita*	Lamiaceae	0.2062	0.3699
Marjoram*	*Origanum jaoranum*	Lamiaceae	0.2076	0.3455
Wormwood*	*Artemisia vulgaris*	Asteraceae	0.2532	0.2858
Broccoli	*Brassica oleraceae*	Brassicaceae	0.0000	1.0000
Wintergreen*	*Gaultheria procumbens*	Ericaceae	0.1654	0.3269
Thyme*	*Thymus vulgaris*	Lamiaceae	0.2054	0.4542
Caraway*	*Carum carvi*	Apiaceae	0.2642	0.2192
Eucalyptus*	*Eucalyptus globulus*	Myrtaceae	0.1658	0.4434
Star Anise*	*Illicium verum*	Schisandraceae	0.2526	0.6972
Oregano*	*Origanum vulgare*	Lamiaceae	0.2070	0.5551
Spearmint*	*Mentha spicata*	Lamiaceae	0.2076	0.8579
Eucalyptus Lemon*	*Corymbia citriodora*	Myrtaceae	0.1651	0.4187
Lemongrass*	*Cymbopogon flexuosus*	Poaceae	0.2427	0.4592
Cinnamon*	*Cinnamomum verum*	Lauraceae	0.1869	0.5986
Garlic*	*Allium sativum*	Amaryllidaceae	0.1779	0.5510
Niaouli*	*Melaleuca quinquenervia*	Myrtaceae	0.1592	0.5250
Cassia	*Cassia auriculata*	Fabaceae	0.1564	0.5127
Rosemary	*Rosmarinus officinalis*	Lamiaceae	0.2003	0.4956
Coriander	*Coriandrum sativum*	Apiaceae	0.2654	0.4406

An asterisk indicates essential oils that were also used in olfactometer assays. ^a^Phylogenetic Distance - calculated using *matK*/*rbcL* chloroplast transcripts. ^b^Physicochemical Similarity – calculated by comparing chemical fingerprints of essential oils to brassica volatiles.

### No-choice tests

Gravid females were introduced singly with two males into 0.3 m^3^ mesh cages (Bioquip, Rancho Dominguez, CA) containing a single broccoli plant treated with an essential oil 48 h prior to exposure. Twenty replicate cages were used for each essential oil treatment. A randomized block design with multiple simultaneous essential oil treatments was not appropriate for these trials because odors from neighboring cages could conceivably alter results in different treatments. Instead, we simultaneously tested a subset of five control plants (sprayed with deionized water), isolated from the treatment, as a check for ovipositional behavior in the absence of non-host odors. Spurious results where control plants had, on average, <5 larvae were discarded and repeated until regular oviposition occurred. Each treatment group spanned two wks of testing (10 treated and five control replicates per wk, N = 20), with at least one wk between each odor to ensure lingering volatiles were cleared from the air in the lab. Cages were placed in a room set at 23 °C with a 16:8 L:D photoperiod using full spectrum fluorescent lights. The ventilation system in the building circulated the air completely every seven mins, so airflow through the mesh cages was consistent.

Due to the small size of midge eggs (<0.5 mm), we were unable to directly count the number of eggs laid. Instead, the infested plants were raised for ~10 d in the UVM greenhouse at 23 °C with a 16:8 L:D photoperiod, so larvae could develop to a size detectable under a dissecting microscope. We processed broccoli plants by excising the growing tip, dissecting the meristem in 70% dilute ethanol, and counting all larvae found on each plant. We tested how larval densities varied between treated and untreated plants using a negative binomial regression in R Studio version 1.2.1335^49^. Figures were generated using the package ggplot2^52^.

### Choice tests

To determine the relative preference between non-host and host odors, we tested if adults (midges) respond differently in assays containing both treated and untreated plants. Each replicate consisted of a cage containing four broccoli plants (8–10 wk old): two treated with 15 ml of a 1% dilution of one of the essential oils and two treated with 15 ml of deionized water. Treated and untreated plants were placed randomly at the corners of a rectangular grid (0.38 m × 0.38 × 0.61 m mesh cages; BioQuip, Rancho Dominguez, CA), matching the minimum commercial spacing for brassica crops^53^ (N = 10). Four gravid female and six male adults were collected from our colony using handheld mouth-aspirators (BioQuip, Rancho Dominguez, CA), released in the center of the cage, and left for 48 h. After the exposure period, we raised the infested plants in the greenhouse at 23 °C with a 16:8 L:D for 10 d, after which we dissected the plants to count larval abundance.

Because the treatments were not independent within the cage, neither parametric nor non-parametric statistics were appropriate to analyze these data^54^. Instead, we used percent change from control from no-choice tests to weight the probability of successfully choosing untreated host plants in choice assays using Hotelling’s T^2^ tests following the F distribution in Microsoft Excel (see Chen and Welter ^55^). In addition, we calculated the odds ratios from a binomial regression using the MASS package in R^56^, to test the likelihood that midges avoid treated plants when untreated plants are present.

### Olfactometer choice tests

In order to determine if swede midge avoids odors from essential oils, we performed behavioral assays using a y-tube olfactometer (Sigma Scientific, Micanopy, FL). We tested the frequency at which mated females (N = 30) avoid host plants treated with one of 15 non-host odors (detailed in Table 1). Rosemary, cassia, and coriander were excluded from olfactometer trials because they were least effective in ovipositional assays. Mated females were allowed 5 min to make a choice between a host plant meristem with ~2 mL of a 1% concentration of an essential oil applied to a #2 medium dental cotton roll and a host plant meristem with a cotton roll treated with water. We tested for the frequency of adult midge responses: (1) choosing the treated arm, (2) choosing the untreated arm, (3) no choice, or (4) stress (adults rolled on their backs and shook their legs). We used binomial exact tests to ask whether non-host essential oils change the expected frequencies (50% choose treated arm:50% choose control arm) of these behaviors for each treatment.

The olfactometer was equipped with two 40-micron mini air filters that remove all liquid and solid particulates. After passing through regulatory components that depressurize incoming air, the airflow is separated into 4 even streams, and passed through individual scrubbing cartridges consisting of acid washed granular activated carbon. Behavioral assays were performed in two 19/22 medium length ground joint glass y-tubes, with 18 mm by 100 mm glass odor chambers attached to either extending arm. Airflow was set at a low rate of 0.1 L/min so midges could travel in the apparatus without being overwhelmed by air velocity^57^, at ~35% RH and 25 ± 2 °C. To control for directional bias, y-tubes were flipped between each replicate. Orientation was a not a significant factor for behaviors so it was excluded from our models. To control for odor dissipation, broccoli tissue/cotton rolls were discarded and replaced after 5 uses. Glassware was wiped clean using Kimwipes and hexane between each treatment.

### Phylogenetic relatedness of plant essential oils

To calculate phylogenetic relatedness, we constructed a phylogeny by concatenating *matK* [gene involved in post-transcriptional processing^58^] and *rbcL* [gene that encodes for rubisco^59^] chloroplast nucleotide sequences for each of the essential oil plant species (https://www.ncbi.nlm.nih.gov/genbank/). These genes were chosen specifically because they are highly conserved chloroplast genes that all plants have. We were able to find sequences in GenBank for both the *matK* or *rbcL* genes for all plant species, except the *rbcL* gene for eucalyptus lemon, so we only used the *matK* gene for this species. Concatenated sequences, excluding eucalyptus lemon, ranged from 1004 (spearmint) to 2180 (rosemary) base pairs in length. Sequence alignments were performed using *muscle*, a command-line application^60^. We used MrBayes^61^, a bioinformatics package that uses Bayesian Markov chain Monte Carlo statistics to build the phylogenetic tree. The sequence alignments were converted to nexus format (required input for MrBayes) using a freely available online tool (http://phylogeny.lirmm.fr). Parameters were set to a GTR nucleotide substitution model with gamma-distributed rate variation across sites. The Bayesian model ran for 2 million generations before convergence on the most supported tree. Finally, we estimated phylogenetic relatedness by calculating the average lengths for each of the branches and summing the distance from each essential oil to the first shared node with *B*. *oleraceae* using FigTree (http://tree.bio.ed.ac.uk/). We tested if there was a relationship between phylogenetic distance, larval infestations in no-choice and choice tests, and olfactometer behavioral trials using negative binomial regressions in RStudio (version 3.2.2). We also used a standard linear regression to estimate R^2^ values for each of these relationships from a linear fit.

### Physicochemical similarity of non-host odors

We calculated physicochemical similarity using ChemMineR, an R package that analyzes the degree of similarity between compounds based on their physicochemical characteristics^62^. The package uses atom path analysis^63,64^ to computationally split compounds into constituent atom pairs and sequences, calculating the lengths of shortest bond paths between atoms. ChemMineR calculates similarity as the fraction of shared atom pairs between user-specified compounds [see Smith *et al*. (1985) for foundational work on the algorithm], following the similar property principle — similar chemical structures should have similar physicochemical properties and biological activities^63^.

Since swede midge are specialized on *B*. *oleraceae*, and odor perception depends on the physical and chemical properties of odors^65^, we compared the odor profiles of each of the essential oils to four isothiocyanate compounds (allyl isothiocyanate, benzyl-isothiocyanate, 3-methylthio-propyl- isothiocyanate, and n-butyl-isothiocyanate) that female midges are known to detect for host acceptance^49^. Compounds present in each of the essential oil treatments were identified using an online database (http://www.nipgr.ac.in/Essoildb/ ^66^) and cross-referenced against PubChem using a python script (Supplemental Appendix 1) that transmitted compound identifiers (ranging from 3–9 digits in length) in exchange for chemical fingerprints^67^.

We compared the compounds using the default Tanimoto similarity coefficient^62–64^, which is part of the ChemmineR pipeline. Values range from 0 (dissimilar) to 1 (identical). We used the averaged physicochemical similarity values between the essential oil compounds and the four brassica isothiocyanates in our models testing whether physicochemical similarity to host odors influences swede midge oviposition or host choice. Poisson regressions were used for ovipositional assays to account for the continuous data and logistic regressions were used for the binary (yes/no) olfactometer data.

## Results

### No-choice and choice tests

**Figure 1:**
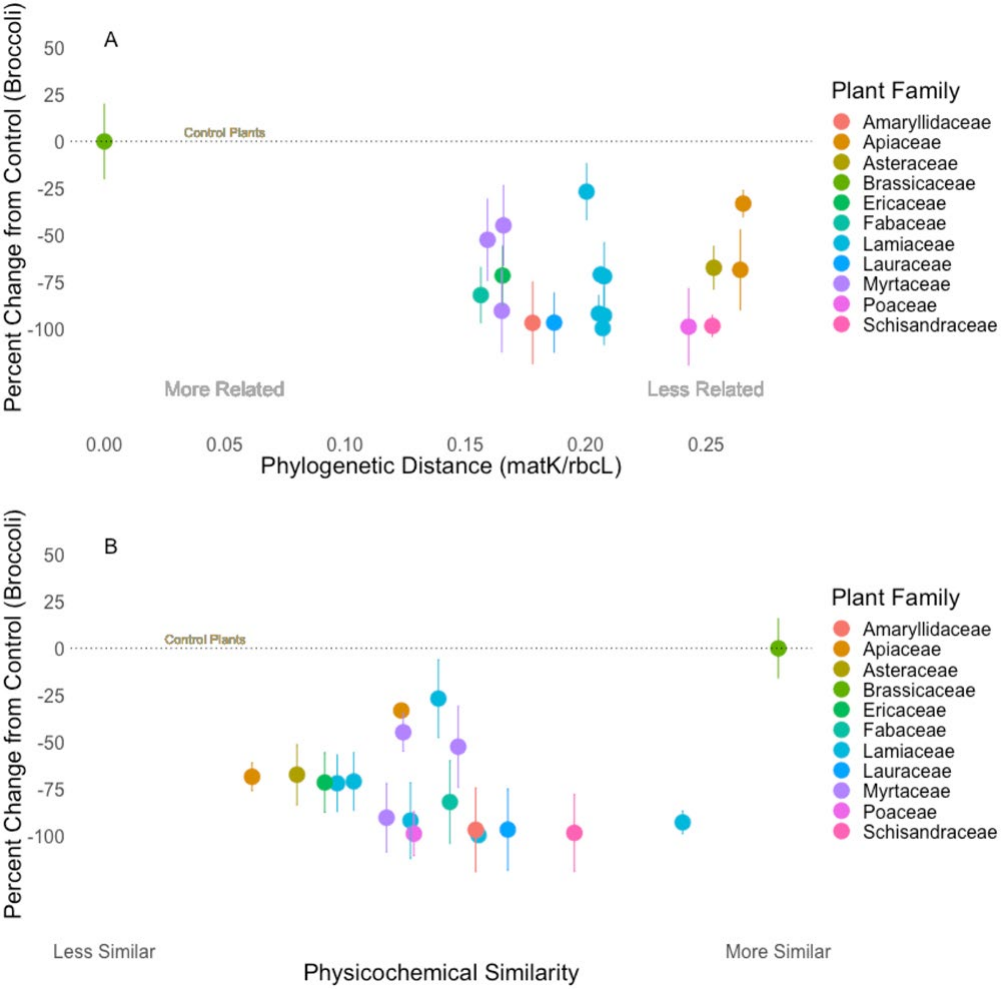
(**A**) Impact of plant essential oils on swede midge (*Contarinia nasturtii*) larval abundance, relative to the control in no-choice tests (N = 20). X-axis values are the phylogenetic distance calculated for each of the essential oils using the concatenated *matK/rbcL* chloroplast sequences (F_19_ = 19.76, R^2^ = 0.47, P < 0.05). (**B**) Impact of plant essential oils on swede midge (*Contarinia nasturtii*) larval abundance, relative to the control in no-choice tests. X-axis is ordered by the physicochemical similarity of the essential oil odors to behaviorally active brassica volatiles (F_19_ = 18.41, R^2^ = 0.45, P < 0.05). Whiskers represent the standard error.

**Figure 2:**
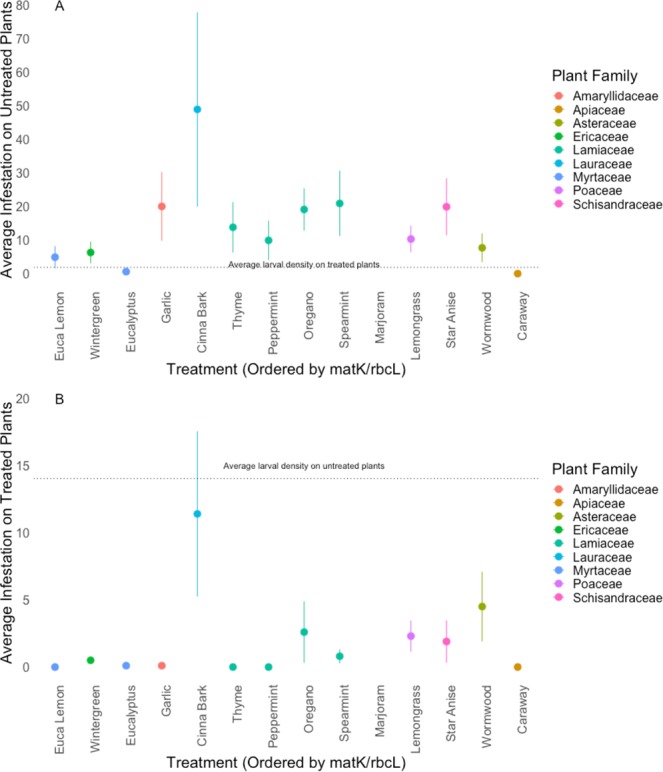
(**A**) Average larval densities of swede midge (*Contarinia nasturtii*) on untreated plants in ovipositional choice assays, containing two treated and two control plants, with treatments ordered by the matK/rbcL phylogeny (N = 10). The dashed line represents mean larval density of treated plants across all replicates. (**B**) Larval densities on treated plants in choice assays ordered by the matK/rbcL phylogeny (N = 10). Dashed line represents mean larval density on untreated plants across all replicates.

**Figure 3:**
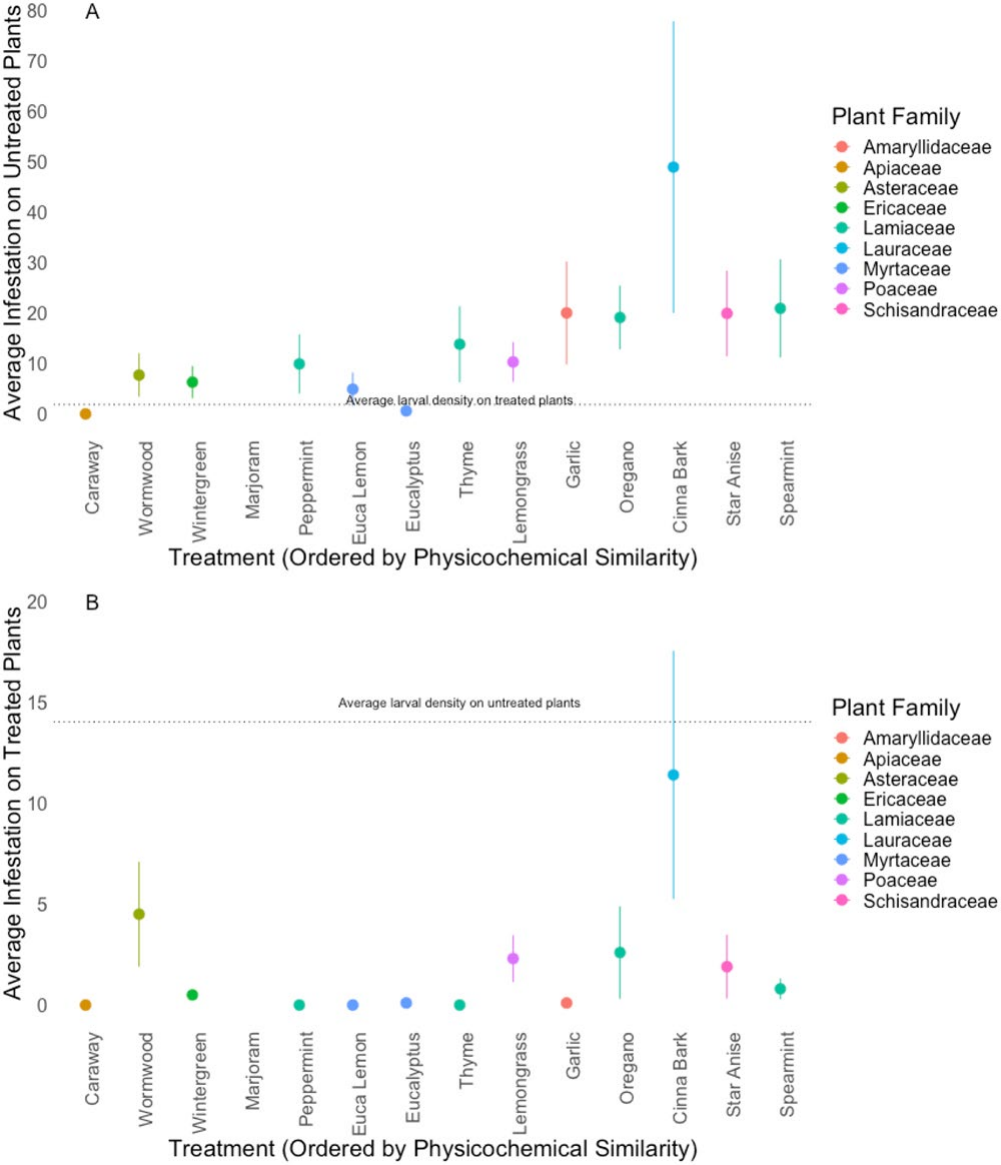
(**A**) Larval densities of swede midge (*Contarinia nasturtii*) on untreated plants in ovipositional choice assays, containing two treated and two control plants, with treatments ordered by physicochemical similarity of the essential oil odors to behaviorally active brassica volatiles (N = 10). The dashed line represents mean larval density of treated plants across all replicates. (**B**) Larval densities on treated plants in choice assays ordered by physicochemical similarity of the essential oil odors to behaviorally active brassica volatiles (N = 10). Dashed line represents mean larval density on untreated plants across all replicates.

Plants treated with essential oils had lower larval densities under no-choice conditions (Fig. 1; *F*_18_ = 5.045, P < 0.05) and were chosen less frequently for oviposition during choice tests (Figs 2 and 3), than those treated with water. While lemongrass, cinnamon, and oregano consistently reduced larval loads, caraway, coriander, and niaouli did not (Fig. 1). Treated plants were, on average, less preferred in choice tests (Table 2, Fig. 2), with cinnamon bark as the exception. While larvae were >1000x less likely to be on plants treated with thyme or eucalyptus lemon compared to untreated plants, they were roughly 5–10x less likely to be found on plants treated with any of the other odors. However, eucalyptus and lemongrass were just as likely to be infested with larvae when caged with untreated plants. Strangely, when caged with both untreated plants and plants treated with cinnamon (a Magnolid in the family Lauraceae; Table 1) midges, on average, oviposited more eggs on both treated and untreated plants than with any other essential oil.

**Table 2:** Swede midge (*Contarinia nasturtii*) choice data. Lower and upper refers to the 95% confidence intervals on the odds ratios from binomial logistic regressions on choice data for each treatment.

Plant	Lower	Upper	Odds Ratio	P
Peppermint	0.598	2.43 × 10^3^	>10^7^	<0.001
Marjoram	0.212	0.987	4	NS
Wormwood	0	1.37	NA	NS
Wintergreen	0.212	1.37	6	NS
Thyme	3.37	2.43 × 10^3^	>10^7^	<0.001
Caraway	NA	NA	NA	NA
Eucalyptus	0.212	0.212	1	NS
Star Anise	0.987	2.68	5	<0.001
Oregano	0.598	2.68	9	<0.001
Spearmint	0.987	1.75	2	<0.001
Eucalyptus Lemon	0.987	2.43 × 10^3^	>10^7^	0.013
Lemongrass	1.37	1.75	1.5	<0.001
Cinnamon	−1.37	−2.18	0.45	<0.001
Garlic	0.212	1.37	6	<0.001

The P values represent the test statistic from Hotelling’ T^2^ tests. Significant P values (P < 0.05) indicate that proportions of larvae on treated versus untreated plants are significantly different. Treated plants were, in general, less preferred by gravid females.

Several essential oil treatments, thyme, star anise, and oregano, had a severe phytotoxic reaction with the broccoli plants, while caraway, coriander, and cinnamon bark were mildly phytotoxic. Since senescing plants are known to release different odors than healthy plants^68^, we tested whether the statistical relationship between essential oil treatments and larval density would remain consistent with phytotoxic treatments removed from the analyses. These tests indicated that the relationships between our independent measures (i.e. phylogenetic relatedness and physicochemical similarity to host odors) and midge behavior were not significantly altered by the phytotoxic treatments (SFig. 1; *F*_13_ = 21.3, R^2^ = 0.5045, P < 0.05 and SFig. 2; *F*_13_ = 19.57, R^2^ = 0.4834, P < 0.05).

### Olfactometer choice tests

**Figure 4:**
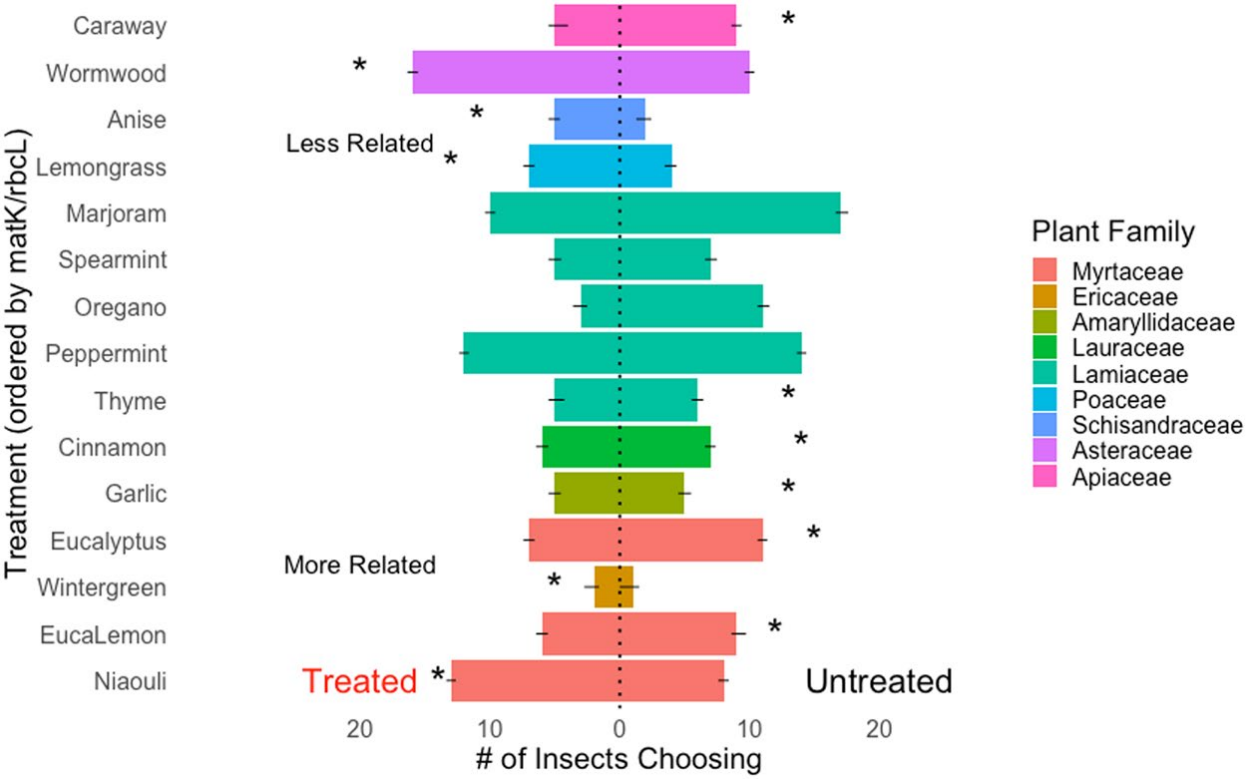
Responses of female swede midge (*Contarinia nasturtii*) to brassica meristems with 1 mL of 1% diluted essential oils (treated) or 1 mL of deionized water (untreated) in olfactometer trials (N = 30). Essential oils (y-axis) are ordered based on phylogenetic distance calculated for each of the essential oils using the concatenated *matK*/*rbcL* chloroplast sequences (z_16_ = 16.05, SE = 1.29, P < 0.05). Asterisks represent significant results from binomial exact tests, with the left indicating that the treated arm was chosen more frequently and the right the control. Whiskers represent standard error.

**Figure 5:**
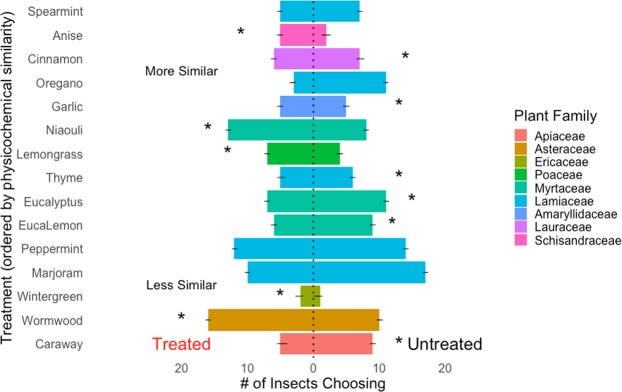
The number (±s.e.) of female swede midge (*Contarinia nasturtii*) responding to brassica meristems with 1 mL of 1% diluted essential oils (treated) or 1 mL of deionized water (untreated) in olfactometer trials (N = 30). Essential oils (y-axis) are ordered based on the physicochemical similarity of the essential oil odors to behaviorally active brassica volatiles (z_16_ = 12.47, SE = 0.85, P < 0.05). Asterisks represent significant results from binomial exact tests, with the left indicating that the treated arm was chosen more frequently and the right the control.

**Figure 6:**
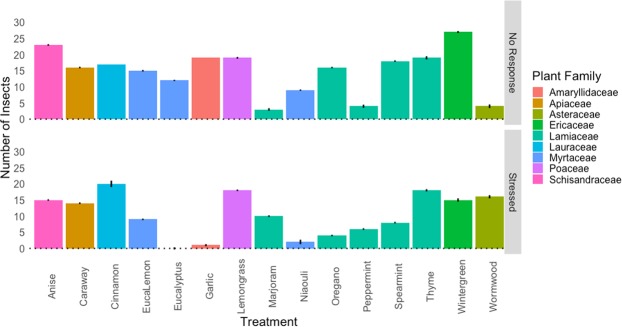
Additional responses of female swede midge (*Contarinia nasturtii*) observed in olfactometer trials (N = 30). Stress refers to cases where midges were agitated by the treatment while no response indicates instances where midges did not make a choice within the 5 m allotted. Whiskers represent standard error.

The majority of the non-host plant essential oils were repellent to the midges. Midges more frequently chose the y-tube arm with the untreated meristems in nearly all treatments. However, there were a few exceptions. When the essential oils from wormwood, wintergreen, niaouli, lemongrass, and star anise were offered as alternatives to untreated broccoli, midges were just as likely to choose the treated arm (significant p-values from binomial exact tests shown on Figs 4 and 5). We also observed that the essential oils caused unanticipated stress responses, suggesting that the odors of essential oils could be toxic as well. For eight trials, the essential oil treatments frequently caused a stress response where midges would roll over and shake their legs. For example, cinnamon bark caused stress in every trial but wormwood, thyme, and lemongrass caused stress in less than half of the replicates (Fig. 6). Phytotoxicity was not a factor in these trials because the odors were applied to cotton rolls rather than host plant tissue.

### Phylogenetic relatedness of plant essential oils

Phylogenetic distance was negatively correlated with larval density in no-choice tests (Fig. 1A; *F*_19_ = 19.76, R^2^ = 0.47, P < 0.05) and olfactometer choice tests (Fig. 4; *z*_16_ = 16.05, SE = 1.29, P < 0.05), meaning that broccoli treated with odors from distantly-related plants had less larvae and were less preferred. Specifically, lemongrass and star anise (distantly related plants from families Poaceae and Schisandraceae, respectively) reduced larval densities by more than 95% and consistently altered host-seeking behavior in y-tubes (Fig. 4). For the more closely related plant families, such as the Malvids, eucalyptus and niaouli (Table 1), essential oils reduced larval density by 44% and 52%. More closely related plant species caused responses that were more varied. In the y-tube, midges more frequently chose meristems in the untreated arm when eucalyptus was used, while the opposite (i.e. midges more frequently chose the treatment) was true with niaouli (Fig. 4).

### Physicochemical similarity of non-host odors

Results from the Poisson regressions indicated that the physicochemical similarity of non-host volatiles to the four behaviorally-active isothiocyanate compounds significantly predicted larval density in no-choice tests (Fig. 1B; *F*_19_ = 18.41, R^2^ = 0.45, P < 0.05). We found that female midges responded similarly in y-tube behavioral trials. Female midges were more likely to make a choice but were as likely to choose the control (Fig. 6; SE = 0.4866, z = 9.000, P < 0.05) or treatment (Fig. 5; SE = 0.4476, z = 9.042, P < 0.05) arms of the y-tube when dissimilar odors were used. When more similar odors were paired with the control, midges were less likely to choose either arm and more likely to not make a choice (Fig. 6). Odors that were more similar came from the plant families Lamiaceae, Lauraceae, or Schisandraceae, such as oregano [physicochemical similarity (PS) = 0.55], cinnamon (PS = 0.59), star anise (PS = 0.69), and spearmint (PS = 0.86). Finally, odors less similar to brassica volatiles, such as caraway (PS = 0.22), wormwood (PS = 0.29), wintergreen (PS = 0.33), and marjoram (PS = 0.35), were also more likely to cause stress in the midges (Fig. 6).

## Discussion

Our results show that essential oils can significantly reduce swede midge larval density and alter female host-seeking behavior. We also found that phylogenetic relatedness and physicochemical similarity significantly influence host choice by this specialist herbivore. However, our study also reveals unexpected results on the nature of repellency. While less phylogenetically related plants are more repellent, distantly-related plants with odors more similar to brassicas [e.g. spearmint (PS = 0.8580) and star anise (PS = 0.6972)] are most repellent. Our work provides a computational approach to understanding repellency.

No previous work has applied phylogenetic relatedness and physicochemical similarity of non-host plants and their odors to repelling a specialist. The insight to measure these characteristics as descriptors of repellency emerged from basic studies on coevolution^57,65,66,69^, chemical ecology^14,70–72^, and the olfactory system^36,71,73,74^, but the accuracy at which these measures captured the most repellent odors was surprising.

Furthermore, while we expected phytochemicals from less phylogenetically related plants to be less physicochemically similar to the isothiocycanate derivatives, this was not the case. Physicochemically similar odors were found throughout the angiosperm phylogeny, with no pattern to their appearance. However, while specific classes of functional groups (e.g. aromatic rings and alkanes) are common across essential oil odors, the entire blends are unique (see Supplemental Figs 2 and 3). The most commonly shared compounds were pinenes, aldehydes, and linalool, with 5–7 different plants having these compounds. Future studies should include tests of other concentrations of essential oils as the amount of any particular compound may also influence its effect on insect behavior. While host recognition and acceptance depends on specific components at particular concentrations from the entire volatile profile^12,13,35,75,76^, it remains unclear whether specialists can even detect the entire bouquet of non-host plants.

It is important to mention some factors that may have influenced the trends observed in our behavioral data. Some of the variation observed between larval densities in ovipositional tests could arguably be due to larvicidal or phytotoxic effects of essential oils^77,78^ rather than repellency. However, our preliminary data indicated that essential oils are not larvicidal (Supplemental Table 1) and, phytotoxicity did not significantly skew our results (Supplemental Figs 1 and 2). Furthermore, the stress response (Fig. 6) observed in olfactometer trials was likely due to fumigant properties^21^. To control for this effect, binomial exact tests of these data excluded replicates where midges were stressed and did not make a choice. Also, it is unclear whether gravid females oviposit immediately on treated plants or wait for the non-host odors to dissipate before contacting broccoli tissue. However, essential oil odors persist for longer than the adult lifespan of the swede midge (Stratton, pers. obs.). Besides, midges in our caged replicates experienced higher concentrations of non-host odors than are naturally released by plants^13,79^. Despite these high concentrations, oviposition still occurred in no-choice tests, and we consistently found significant differences in larval abundance on treated and untreated plants in choice tests.

In a few olfactometer treatments, midges more frequently chose the non-host odors, but unexpectedly, these odors also consistently caused a stress response. For example, females preferred to move towards *Artemisia vulgaris* (wormwood), *Cymbopogon flexuosus* (lemongrass), and *Illicium verum* (star anise) they caused stress in more than half of the females (Figs 5 and 6). The volatile profile of wormwood, lemongrass, and star anise consist of 8, 8, and 9 compounds, respectively, and while wormwood and lemongrass both contain myrcene, the rest of the odor profiles are unique. Physicochemically, these volatile profiles each differ from brassica volatiles by 0.29, 0.46, and 0.61 (Table 1), so it is unclear why these odors had a similar effect on female midges in our assays. It is possible that a portion of the essential oil volatiles synergistically enhanced the reception of host odors^80–82^ while the other compounds caused a delayed stress response^77^. It would be interesting to test combinations of these compounds for their ability to attract and potentially kill gravid swede midge.

Plant essential oils are diverse in functional groups and effective products against target insects^21,77,83^. Future work should test specialists of other plants and generalists of multiple plants for comparison with how phylogenetic relatedness and physicochemical similarity influence insect behavior. Predicting repellency may be a complex task, as related insects are seldom repelled by the same compounds. For example, while swede midge and hessian fly (*Mayetiola destructor*; Diptera: Cecidomyiidae) are both gall midges, eucalyptus repels hessian fly^21,25^ with moderate toxicity^84^, but was relatively inert for swede midge (Figs 1, 4–6). Our phylogenetic and physicochemical measures could be modified to use wheat as the focal plant, but behavioral datasets would be required to test our questions in any additional systems. For swede midge, essential oils such as garlic or eucalyptus lemon appear most promising for field testing as repellents for pest management because these odors reduced larval densities without phytotoxic effects (Fig. 1).

We show that specialist herbivores can clearly respond to unfamiliar odors in their environment, and their behavioral responses may be predictable. Using repellent plant compounds is a promising avenue that can be exploited for sustainable pest management; however, further work is needed to guide the selection of repellent odors for specialist herbivore pests. With the development of systematic techniques to test and compare how non-host odors influence pest insect behavior, the odors of agricultural landscapes could be altered to repel (or attract) specific insects. Plant essential oils are an important tool to consider for sustainable pest management^21,25,77^, for which we have barely scratched (and sniffed) the potential.

## Supplementary information

Supplementary Materials

## Data Availability

All data generated or analyzed during this study are included in this published article (and its Supplementary Information Files).
