# Supplementary material for: Odors from phylogenetically-distant plants to Brassicaceae repel an herbivorous Brassica specialist
Source: Sci Rep. 2019 Jul 23;9:10621. doi: 10.1038/s41598-019-47094-8 (PMC6650400; doi:10.1038/s41598-019-47094-8)
Supplement: Supplementary file 1 — Supplementary Materials [file 41598_2019_47094_MOESM1_ESM.docx]

C. A. Stratton

Department of Plant and Soil Sciences

University of Vermont

63 Carrigan Dr.

Burlington, VT 05405

Phone: (804) 397-5720

E-mail: castratt@uvm.edu

SUPPLEMENTARY MATERIALS

Odors from phylogenetically-distant plants to Brassicaceae repel an herbivorous Brassica specialist

AUTHORS

Chase A Stratton^1^, Elisabeth Hodgdon^1^, Cesar Rodriguez-Saona^2^, Anthony M Shelton^3^, and Yolanda H Chen^1^

^1^Department of Plant and Soil Sciences, University of Vermont, 63 Carrigan Dr, Burlington, VT, 05405
^2^ Department of Entomology, Rutgers The State University of New Jersey, 96 Lipman Dr, New Brunswick, NJ, 08901
^3^Department of Entomology, Cornell University, New York State Agricultural Experiment Station, 630 West North St, Geneva, NY, 14456

**Supplemental Methods, Figures, and Appendix**

*Phytotoxicity of Essential Oils*

 A few of the essential oil treatments had a severe phytotoxic reaction with broccoli plants, literally melting the tissue during caged ovipositional assays. Thyme, star anise, and oregano caused the most severe reaction while caraway, coriander, and cinnamon bark caused a milder reaction with only minor scarring on broccoli plants. To account for the possibility that the odors released by senescing plants were influencing swede midge oviposition rather than the essential oils, we performed additional statistical tests excluding data from phytotoxic essential oils. Phylogenetic relatedness (SFig1; *F*_13_ = 21.3, R^2^ = 0.5045, P < 0.05) and physicochemical similarity (SFig2; *F*_13_ = 19.57, R^2^ = 0.4834, P < 0.05) of non-host odors to brassicas remained significant predictors of larval density from these additional models.


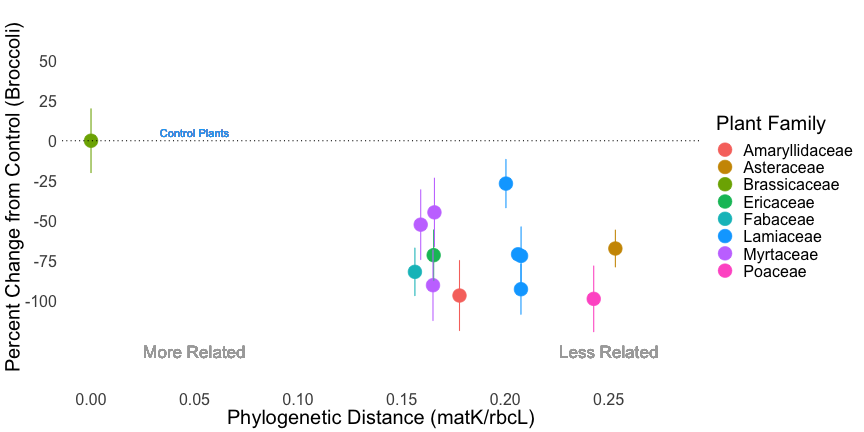


Supplemental Figure 1 - Impact of non-phytotoxic plant essential oils on swede midge (*Contarinia nasturtii*) larval abundance, relative to the control in no-choice tests. X-axis values are the phylogenetic distance calculated for each of the essential oils using the concatenated matK/rbcL chloroplast sequences (*F*_13_ = 21.3, R^2^ = 0.5045, P < 0.05). Whiskers represent the standard error.


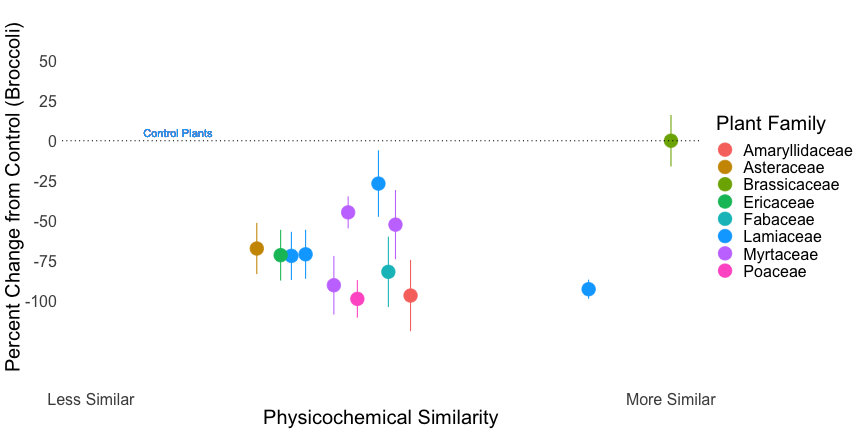


Supplemental Figure 2 - Impact of non-phytotoxic plant essential oils on swede midge (Contarinia nasturtii) larval abundance, relative to the control in no-choice tests. X-axis is ordered by the physicochemical similarity of the essential oil odors to behaviorally active brassica volatiles (*F*_13_ = 19.57, R^2^ = 0.4834, P < 0.05). Whiskers represent the standard error.

*Larval mortality*
Supplemental Table 1 – Summary statistics for a twelve-replicate experiment testing for larvicidal effects of essential oil treatments on swede midge. Larvae were applied to broccoli, grown using the same methods described in the results, using a protocol described in Stratton et al. (2018). Following the inoculation procedure, the essential oils (or water for control) were applied to 4 plants following the protocol for no-choice assays. Since we were not testing the effect of odors on behavior, this experiment allowed for a randomized block design. Importantly, our previous work found that, on average, half the larvae perish following the inoculation procedure. These trials indicated that essential oils were not larvicidal.

| Essential Oil | Replicates | # Larvae Applied | Average # Larvae Survived ± Standard Deviation |
| --- | --- | --- | --- |
| Garlic | 12 | 30 | 17.083 ± 8.929 |
| Lemongrass | 12 | 30 | 12.500 ± 5.854 |
| Wintergreen | 12 | 30 | 14.000 ± 8.613 |
| Control | 12 | 30 | 16.250 ± 8.625 |


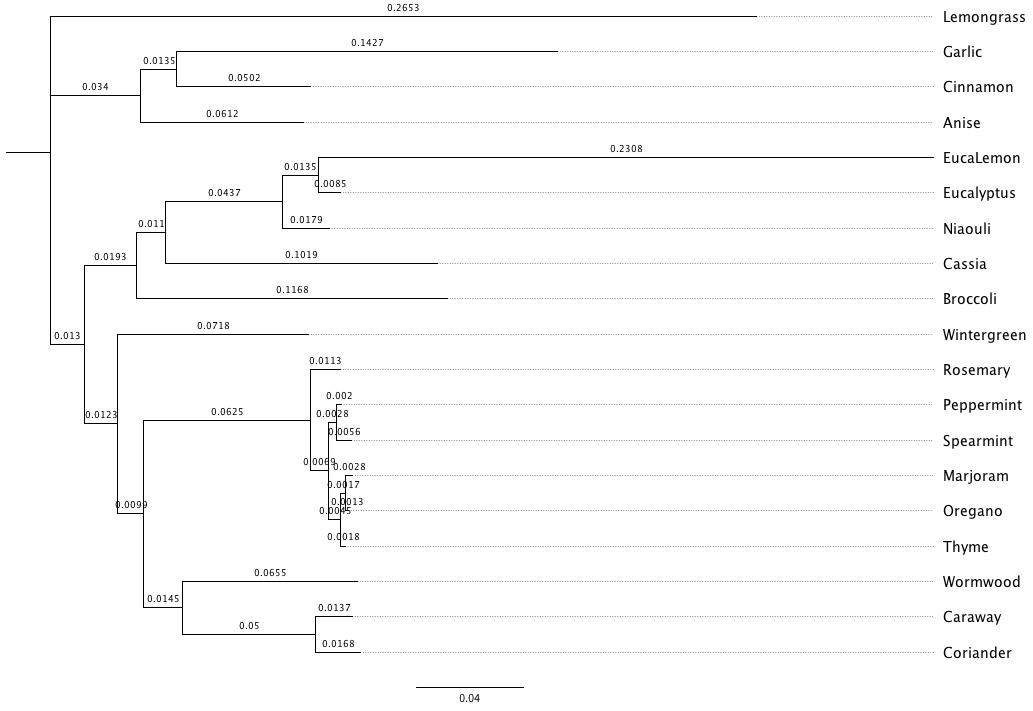

Supplemental Figure 3 – Phylogenetic tree with raw branch lengths that were used to estimate phylogenetic relatedness values for each of the plant essential oils. Since species within a phylogeny are not independent, average lengths were calculated for each branching event, then summed to a shared node between an essential oil and broccoli.


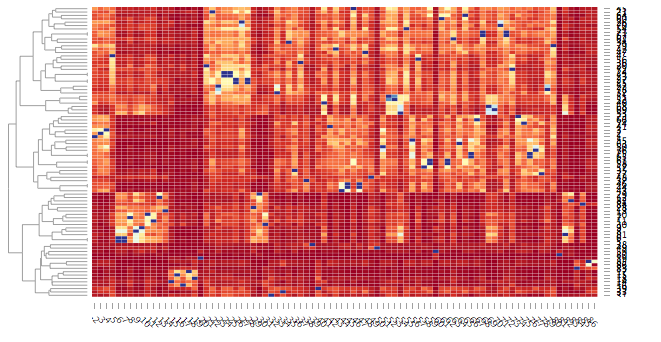


Supplemental Figure 4 – D3 heatmap^1^ showing similarity values between all essential oil compounds, calculated using ChemMineR^2^. Blue cells indicate compounds that are physicochemically similar while red indicate compounds that differ. On the left of the heatmap is a chemical dendrogram emphasizing the groupings that occur in plant chemistries.


*Gas chromatography*

 To confirm that the commercial essential oils varied in volatile properties, volatile collections were performed on 0.01% dilute concentrations of essential oils using a push-pull system described in Szendrei et al. (2009). Purified air entered the odor chamber at 0.2 L/min and volatiles were collected in Alltech Super-Q absorbent traps. Volatiles were eluted from the traps using dichloromethane (150 μL) and 400 ng of n-octane was used as an internal standard. Compounds were separated and quantified using a Hewlett-Packard 6890 Series gas chromatograph with a flame ionization detector (GC-FID). Helium was used as the carrier gas and the temperature program was set at 40°C for 1 min, 14°C/min to 180°C, and 40°C/min to 200°C. After quantifying the individual compounds present in each essential oil, we selected for the most prominent peaks in each of the chromatographs and extracted the raw data for those retention times. Supplemental figure 3 shows the magnitude of variation in essential oil volatile chemistries.


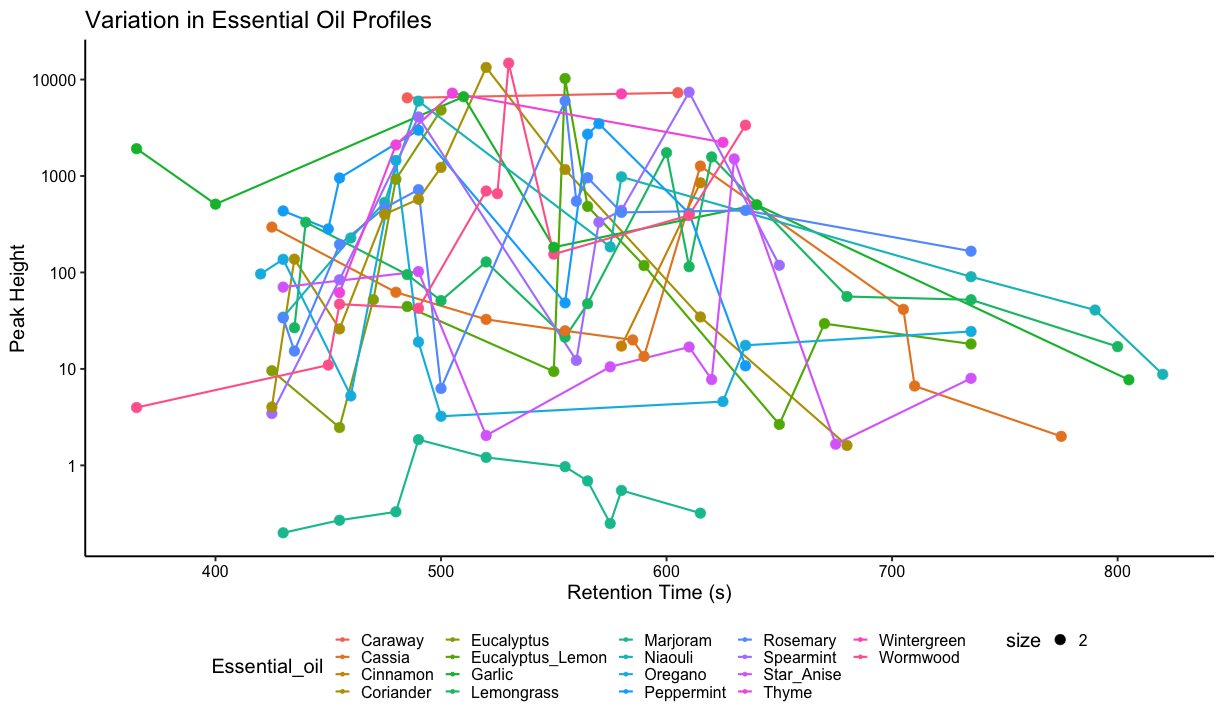


Supplemental Figure 5 – Log-transformed peak height at retention times ranging from ~350-820 s. Larger molecules take longer to reach the machines flame ionization detector so have higher retention times. Peak height roughly estimates to the amount of compounds captured at any particular retention time.

**Appendix 1** – Python script for querying PubChem database for CIDs


#!/usr/bin/env python

#This program will use compound names in a .csv file to search PubChem and retrieve the CID values

#Loading the necessary modules

import pubchempy as pcp

import pandas as pd

import re

inFile = "~/path/to/chemical/file/pubchem-query.csv"

df = pd.read_csv(inFile)

compounds = df.Compound

#cidSet = pcp.get_compounds(compounds,'name')

cidSet = []

print(cidSet)

for i in range(0,X): #Replace X with the number of compounds in .csv file

cidSet = pcp.get_compounds(compounds[i],'name')

print(cidSet)

Works Cited

1. Bostock, M., Ogievetsky, V. & Heer, J. D^3^ Data-Driven Documents. *IEEE Trans. Vis. Comput. Graph.* **17**, 2301–2309 (2011).

2. Cao, Y., Charisi, A., Cheng, L. C., Jiang, T. & Girke, T. ChemmineR: A compound mining framework for R. *Bioinformatics* **24**, 1733–1734 (2008).

3. Szendrei, Z., Malo, E., Stelinski, L. & Rodriguez-Saona, C. Response of Cranberry Weevil (Coleoptera: Curculionidae) to Host Plant Volatiles. *Chem. Ecol.* **38**, 861–869 (2009).
